# Meta-Analysis of Fecal Microbiota and Metabolites in Experimental Colitic Mice during the Inflammatory and Healing Phases

**DOI:** 10.3390/nu9121329

**Published:** 2017-12-06

**Authors:** Toshifumi Osaka, Eri Moriyama, Shunichi Arai, Yasuhiro Date, Junji Yagi, Jun Kikuchi, Satoshi Tsuneda

**Affiliations:** 1 Department of Microbiology and Immunology, Tokyo Women’s Medical University, 8-1, Kawada-cho, Shinjuku-ku, Tokyo 162-8666, Japan; osaka.toshifumi@twmu.ac.jp (T.O.); yagi.junji@twmu.ac.jp (J.Y.); 2 Department of Life Science and Medical Bioscience, Waseda University, 2-2 Wakamatsu-cho, Shinjuku-ku, Tokyo 162-8480, Japan; e.moriyama2112@gmail.com (E.M.); s_arai@ruri.waseda.jp (S.A.); 3 RIKEN Center for Sustainable Resource Science, 1-7-22 Suehirocho, Tsurumi-ku, Yokohama, Kanagawa 230-0045, Japan; yasuhiro.date@riken.jp (Y.D.); jun.kikuchi@riken.jp (J.K.); 4 Graduate School of Medical Life Science, Yokohama City University, 1-7-29 Suehirocho, Tsurumi-ku, Yokohama, Kanagawa 230-0045, Japan; 5 Graduate School of Bioagricultural Sciences, Nagoya University, 1 Furo-cho, Chikusa-ku, Nagoya, Aichi 464-0810, Japan

**Keywords:** gut microbiota, dysbiosis, inflammatory bowel disease, metabolome, meta 16S rRNA analysis, ^1^H-NMR analysis, experimental colitic mice, *Lactobacillus*

## Abstract

The imbalance of gut microbiota is known to be associated with inflammatory bowel disease, but it remains unknown whether dysbiosis is a cause or consequence of chronic gut inflammation. In order to investigate the effects of gut inflammation on microbiota and metabolome, the sequential changes in gut microbiota and metabolites from the onset of colitis to the recovery in dextran sulfate sodium-induced colitic mice were characterized by using meta 16S rRNA sequencing and proton nuclear magnetic resonance (^1^H-NMR) analysis. Mice in the colitis progression phase showed the transient expansions of two bacterial families including Bacteroidaceae and Enterobacteriaceae and the depletion of major gut commensal bacteria belonging to the uncultured Bacteroidales family S24-7, Rikenellaceae, Lachnospiraceae, and Ruminococcaceae. After the initiation of the recovery, commensal *Lactobacillus* members promptly predominated in gut while other normally abundant bacteria excluding the Erysipelotrichaceae remained diminished. Furthermore, ^1^H-NMR analysis revealed characteristic fluctuations in fecal levels of organic acids (lactate and succinate) associated with the disease states. In conclusion, acute intestinal inflammation is a perturbation factor of gut microbiota but alters the intestinal environments suitable for *Lactobacillus* members.

## 1. Introduction

Inflammatory bowel disease (IBD), which includes Crohn’s disease and ulcerative colitis, is characterized by chronic and relapsing inflammation of the gut. Although the definite pathogenesis of IBD remains unclear, numerous studies have suggested the genetic and environment factors associated with chronic intestinal inflammation. Meta-analysis of genome-wide association studies have discovered many IBD susceptibility genes including cytokine regulation, innate immunity, and lymphocyte activation [1]. In many immune-deficient mice that spontaneously develop colitis, the presence of gut commensal bacteria is essential for the development of intestinal inflammation [2,3,4]. A recent molecular phylogenetic analysis revealed the imbalanced bacterial composition (dysbiosis) in human IBD patients [5,6,7] and IBD model animals [8,9]. However, it is not clear whether dysbiosis is actually a cause or consequence of dysregulated mucosal immune response in IBD.

Gut environments including microbiota and its metabolites influence the maintenance of gut homeostasis. Short chain fatty acids (SCFAs, e.g., acetate, butyrate, and propionate), which are produced by gut commensal bacteria from dietary fiber, have profound effects on energy metabolism, hormone production, intestinal epithelial homeostasis, mucosal immunity, and virulence regulation of pathogens [10,11]. Mice lacking SCFA receptors such as GPR43 and GPR109a are highly susceptible to chemical-induced colitis [12,13]. Among SCFAs, butyrate produced by gut commensal bacteria plays an essential role in the induction of regulatory T cell (Treg), which is involved in immunosuppressive mechanisms in the colon [14,15]. Furthermore, microbiome studies in IBD patients have reported the reduction in these Treg-inducing bacterial population including *Clostridia* clusters IV and XIVa [5,6]. These findings indicate the possibility that dysbiosis-induced changes in gut metabolites are responsible for the pathogenesis of IBD. 

The manipulation of gut microbiota with antibiotics administration [16,17], probiotics administration [18,19,20], or fecal microbiota transplantation (FMT) [21,22,23] provides prospective therapeutic options for IBD. As with any medication, these therapies come with potential risks and side effects [24,25,26]. Several studies of FMT application have reported the risk of relapse or worsening of gut inflammation [25,26] and the development of obesity [27]. Antibiotic treatment has also some concerns such as the onset of *Clostridium difficile* infection [28] and the overgrowth of mucosal-associated bacteria after its cessation [29]. Thus, accumulating knowledge of how dysbiosis is developed in IBD patients is required for disease control of IBD over the long-term period.

This study aimed to characterize the dynamics of gut microbiota and metabolites from the onset of colitis to the recovery period by using a multi-omics approach consisting of meta 16S rRNA sequencing and proton nuclear magnetic resonance (^1^H-NMR) analysis. We used a dextran sulfate sodium (DSS)-induced colitis model. Mice given DSS exhibit reproducibly acute colitis, while mice gradually convalesce after its removal. This feature is useful for characterizing the relationships between gut environments and intestinal inflammation. We demonstrate here characteristic dynamics of commensal bacteria and organic acids associated with disease states.

## 2. Materials and Methods

### 2.1. Animal Experiment

Six-week-old female C57BL/6J mice (*n* = 6) were obtained from CREA Japan, Inc. (Tokyo, Japan) and co-housed for 2 weeks before the start of DSS administration. For chemical-induced colitis, mice were fed with 5% DSS (molecular weight: 5000 Da; Wako, Osaka, Japan) ad libitum for 5 days. Then, mice were given regular water in the remaining periods. Body weight and bleeding score (no bleeding, 0; bleeding, 1; gross bleeding, 2) were recorded. A total of 46 fecal samples were collected on days 0, 5, 6, 8, 9, 11, 13, 15, and 20 for the gut microbiome and metabolome analysis. Animal experiments in this study were approved by the ethical committee of Waseda University Academic Research Ethics Committee (2013—A073a, 2014—A035a, 2015—A066).

### 2.2. 16S rRNA Gene-Based Microbiome Profiling

We extracted the DNA from fecal samples using ISOFECAL for Beads Beating (Nippon Gene, Inc., Toyama, Japan). The V1-2 region of 16S rRNA gene was amplified using the 27F/357R primer set [30] with the Ion Xpress Barcode Adaptor sequences. The concentration and fragment size of PCR amplicons purified using a Wizard SV Gel and PCR Clean-Up System (Promega, Madison, WI, USA) were determined using an Agilent 2100 Bioanalyzer (Agilent Technologies, Palo Alto, CA, USA). The diluted pooled amplicons were clonally amplified by emulsion PCR and enriched template-positive particles using an Ion OneTouch 2 instrument (Life Technologies, Carlsbad, CA, USA) and an Ion PGM Template OT2 400 Kit (Life Technologies) according to their instructions. Sequencing was carried out using an Ion Sequencing 400 kit and Ion 314 chip (Life Technologies) on the Ion PGM system. Sequence data were processed using QIIME 1.8.0 [31] to determine the operational taxonomic unit (OTU) as the same phylotype at 97% identity threshold.

### 2.3. Fecal Metabolites Analysis Using ^1^H-NMR Analysis

The freeze-dried and grounded feces were extracted by mixing with 100 mM potassium phosphate buffer in D_2_O (pH 7.4) containing 1 mM sodium 2,2-dimethyl-2-silapentane-5-sulfonate as internal standard. After centrifugation, the supernatants of water-soluble components were collected for NMR measurements with AVANCE II 700 MHz Bruker Biospin (Bruker, Rheinstetten, Germany) as described previously [32,33,34,35]. The NMR spectra were processed using TopSpin 3.1 software (Bruker) and each of the peak signal intensities were calculated. For statistical analysis using R software [36], the spectra data were reduced by subdividing spectra into sequential 0.02 ppm. As mentioned in previous reports [37,38,39,40,41], the NMR peaks of interest were assigned using two web databases (SpinAssign, HMDB) with assistance of 2-dimension NMR spectra (^1^H-^13^C heteronuclear single quantum coherence) data of fecal samples in DSS-treated mice, which was collected in another research project.

### 2.4. Measurements of Lactate and Succinate in Fecal Samples

Fecal samples collected from five mice were suspended in distilled water. After homogenization with a handy homogenizer NS-310EIII (Microtech Corp., Chiba, Japan), fecal suspensions were heated for 15 min at 50 °C in a water bath, and filtered through a 0.2-μm cellulose acetate syringe filter (Advantech, Tokyo, Japan). Fecal extracts were mixed with an equal volume of chloroform, and centrifuged at 15,000 rpm for 10 min. The aqueous phase was mixed with two volumes of acetonitrile, and centrifuged at 15,000 rpm for 10 min. For succinate analysis, the aqueous phase was further purified with nine volumes of acetonitrile. After centrifugation, lactate and succinate in the deproteinized supernatants were measured with an ion chromatography ICS2100 (Thermo Fisher Scientific Inc., Waltham, MA, USA) and a liquid chromatography electrospray quadrupole time-of-flight mass spectrometry Xevo G2-XS QTof (Waters Corp., Milford, MA, USA).

### 2.5. Statistical Analysis

Multidimensional scaling (MDS) of fecal microbiome and principal component analysis (PCA) of fecal metabolome were performed using the R software package, ver 2.15.3 [36]. Using the statistical software JMP^®^ 12 (SAS Institute Inc., Cary, NC, USA), the correlations between microbiome and metabolites were screened, and analyses of variance with a post hoc Turkey-Kramer honestly were performed to identify significant differences in relative abundance of bacterial taxa or peak signal intensities of metabolites among the pathological phase.

## 3. Results

### 3.1. Clinical Manifestation of DSS-Treated Mice

**Figure 1:**
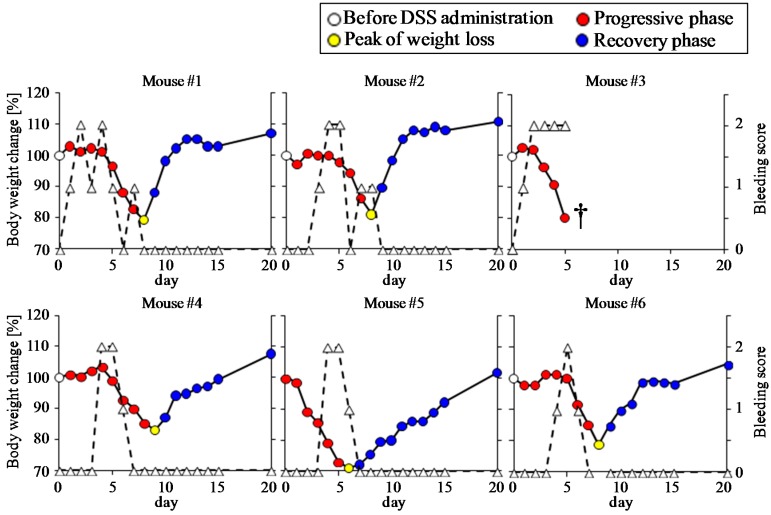
Monitoring of body weight changes and bleeding score of dextran sulfate sodium (DSS)-treated mice. Body weight changes (solid lines) are expressed as the percent of initial body weight. Bleeding (dashed lines) in mice was scored according to the following criteria: no bleeding, 0; bleeding, 1; gross bleeding, 2. The disease phases were categorized according to body weight changes during the experimental period: before DSS administration (phase 1, white), progressive phase (phase 2, red), a peak of weight loss (phase 3, yellow), recovery phase (phase 4, blue). One of the mice (No. 3) died on day 5 (†).

Oral administration of 5% DSS for 5 days was carried out to induce colitis in wild type C57BL/6 mice (*n* = 6). The body weight began to decrease 2 to 6 days after DSS administration (Figure 1). Two mice (No. 3 and No. 5) that exhibited high sensitivity to DSS showed a drastic decrease in body weight. One of these mice (No. 3) died on day 5 due to massive bleeding. For the remaining five mice, the bleeding disappeared and the body weight began to increase 1 to 4 days after the removal of DSS from the drinking water. The periods required for the mice to recover to their initial body weights ranged between 3 to 14 days after the removal of DSS. The body weight loss was well-recognized as the primary indicator of DSS-induced colonic damage. Thus, we categorized the disease phases according to body weight changes during the experimental period: before DSS administration (phase 1), progressive phase (phase 2), a peak of weight loss (phase 3), recovery phase (phase 4).

### 3.2. Time-Course Transitions in Colonic Bacterial Compositions in DSS-Treated Mice

**Figure 2:**
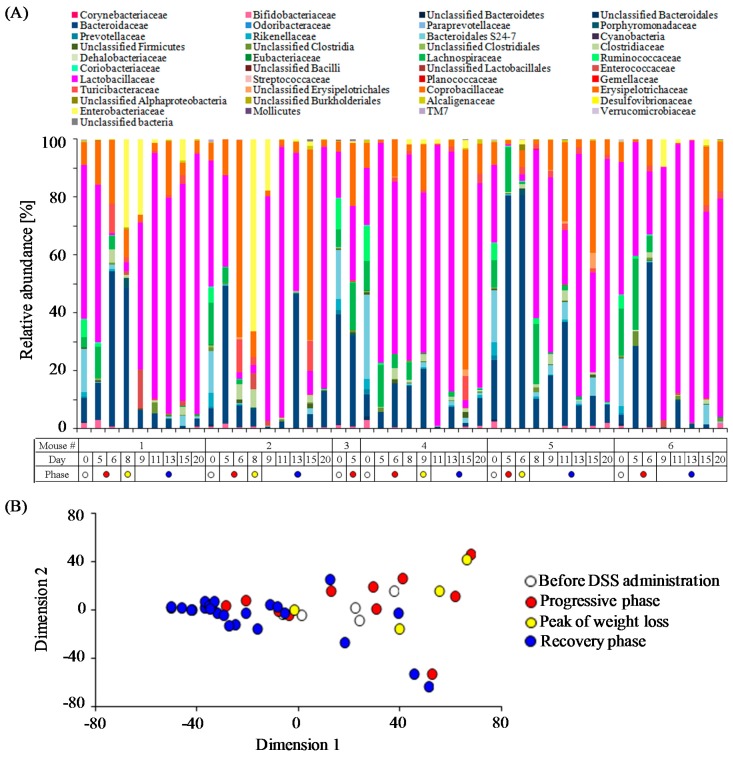
Fecal microbiome profiles during the experimental periods. (**A**) Changes in the relative abundance at the family level in each DSS-treated mouse; (**B**) Multidimensional scaling (MDS) plot of fecal microbiome (at the family level) at each time point.

The colonic bacterial community compositions in each mouse during the experimental period were monitored by 16S rRNA gene (V1–V2 regions) amplicons sequencing. A total of 147,794 sequences (an average of 3213 sequences per sample) obtained from 46 fecal samples were clustered into 2625 OTUs. Time-course changes in relative abundance of taxa classified at the family level within individual DSS-treated mouse are shown in Figure 2A. Prominent bacterial members in DSS-untreated mice, corresponding to the data at day 0 in Figure 2A, included members of eight families: Lactobacillaceae (34.1 ± 6.3%), uncultured Bacteroidales family S24-7 (18.3 ± 2.2%), Bacteroidaceae (13.8 ± 5.4%), Lachnospiraceae (10.0 ± 2.0%), Ruminococcaceae (7.4 ± 1.3%), Erysipelotrichaceae (6.8 ± 0.8%), Rikenellaceae (3.5 ± 0.5%), and Bifidobacteriaceae (1.8 ± 0.4%). Notably, the abundance of the Bacteroidaceae in the two mice with high sensitivity to DSS (NO.3, 37.9%; NO.5, 20.6%) was much higher than the other four mice (3.2% to 8.1%). 

**Figure 3:**
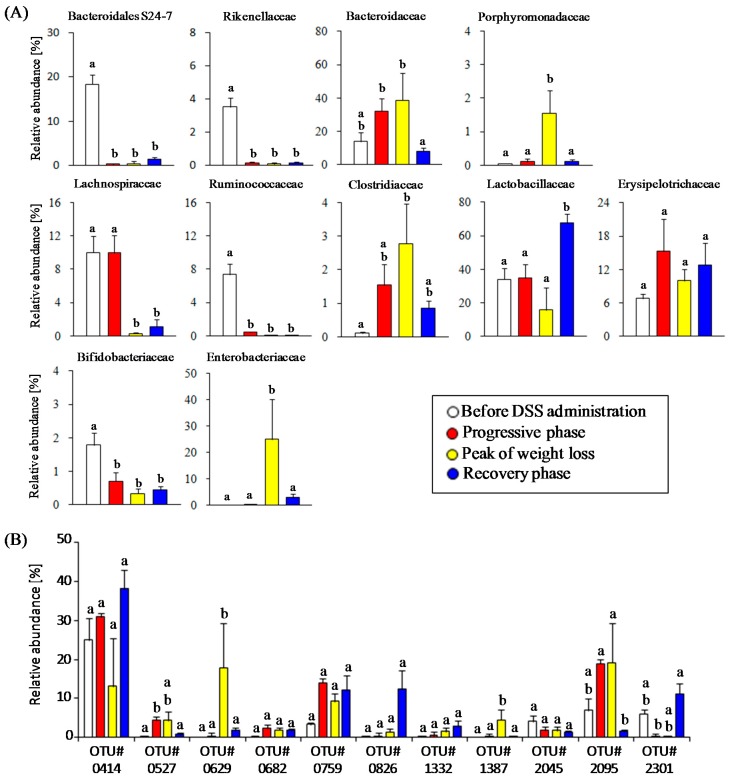
Characteristics of changes in relative abundances of major commensal bacterial groups. (**A**) Changes in the abundances of eleven bacterial families in four pathological phases; (**B**) Changes of the top five most abundant operational taxonomic units (OTUs) in each phase. Different letters indicate significant differences between bars.

To characterize the time-course transitions in microbiota from the onset of colitis to the recovery phase, we performed a MDS analysis. The MDS plot of bacterial community composition showed a gut microbiota disturbance by the treatment with DSS (Figure 2B). Next, we characterized the relative abundance in eleven bacterial families in each phase after DSS administration. The abundances of the Bacteroidaceae (38.7 ± 16.5%), Enterobacteriaceae (25.0 ± 15.4%), Clostridiaceae (2.8 ± 1.2%), and Porphyromonadaceae (1.6 ± 0.7%) increased in the phase of colitis progression but rapidly decreased in the recovery phase (Figure 3A). On the other hand, the onset of DSS-induced colitis resulted in a significant decrease in the abundances of Bifidobacteriaceae (0.7 ± 0.4%), uncultured Bacteroidales family S24-7 (0.5 ± 0.5%), Rikenellaceae (0.1 ± 0.1%), Lachnospiraceae (0.3 ± 0.1%), and Ruminococcaceae (0.03 ± 0.03%). Furthermore, these four bacterial families did not increase in the recovery phase. The abundances of two bacterial families (i.e., Lactobacillaceae, Erysipelotrichaceae) did not change during the colitis progression, but the Lactobacillaceae increased beyond the baseline level immediately after weight gain in the recovery phase. Taken together, our results show the characteristic population changes of bacterial families associated with the development and termination of intestinal inflammation. 

Furthermore, we identified abundant bacterial phylotypes in each phase. When viewed at the OTU level, eleven OTUs were found in the top five most abundant OTUs in four phases (Table 1). Changes in the relative abundances of these OTUs among the disease phases are shown in Figure 3B. The OTU414 (*Lactobacillus johnsonii*-related 16S rRNA sequence) was most abundant in all of OTUs detected in this study. The relative abundance of the OTU414 showed an increasing tendency after DSS treatment, but this was not statistically significant. On the other hand, the relative abundance of other two OTUs (OTU826 and OTU2301) affiliated with the genus *Lactobacillus* during the experiment period showed different trends from the OTU414. Treatment with DSS resulted in a rapid decrease in the abundance of the OTU2301 (*L. intestinalis*), and then the abundances of OTU826 (*L. murinus*) and OTU2301 were dramatically elevated in the recovery phase. DSS-induced colitis led to a transient overgrowth during the weight-loss period in the following OTUs: OTU527 (*Bacteroides uniformis*), OTU629 (*Escherichia coli*), OTU1387 (*E. coli*), and OTU2095 (*B. acidifaciens*). Unlike the above *Bacteroides*-related OTUs, the OTU2045 (*Bacteroides* sp.) was slightly reduced after DSS treatment. Three phylotypes (OTU682, OTU759, and OTU1332) tended to increase after the onset of colitis, and then remained stable during the experiment period. Thus, we screened the abundant bacterial phylotypes that responded to gut environmental changes induced by acute colitis.

### 3.3. Characterization of Fecal Metabolites in DSS-Treated Mice

**Figure 4:**
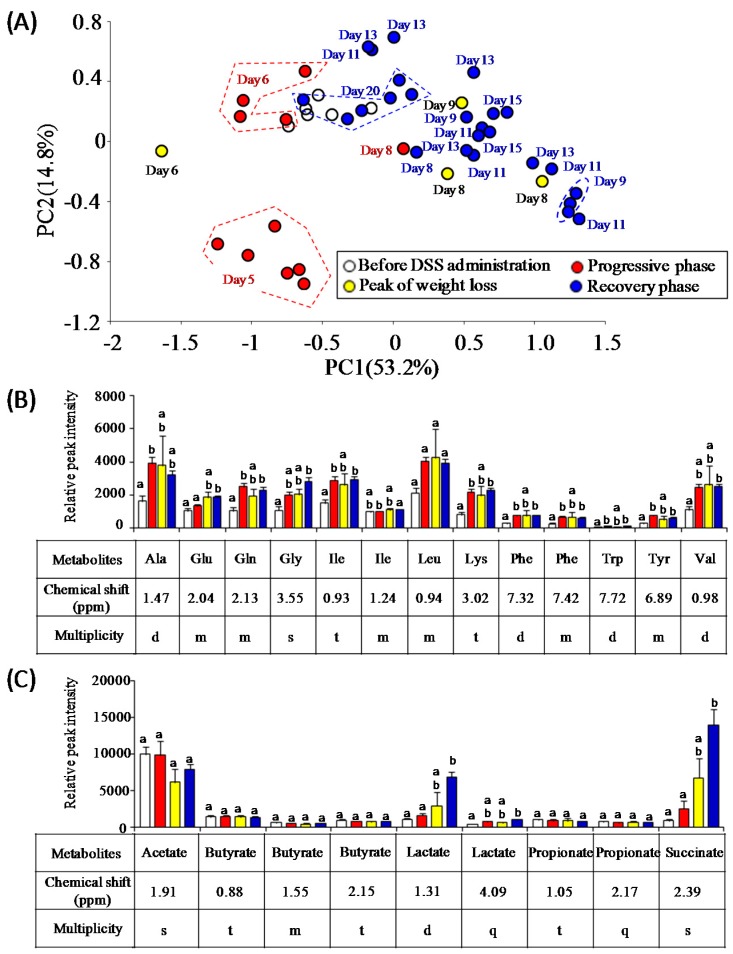
Characteristics of changes in fecal metabolites of DSS-treated mice. (**A**) Principal component analysis (PCA) score plots of fecal metabolites at each time point; (**B**) Changes in relative peak intensities of amino acids; (**C**) Changes in relative peak intensities of short chain fatty acids and organic acids. Different letters indicate significant differences between bars.

**Figure 5:**
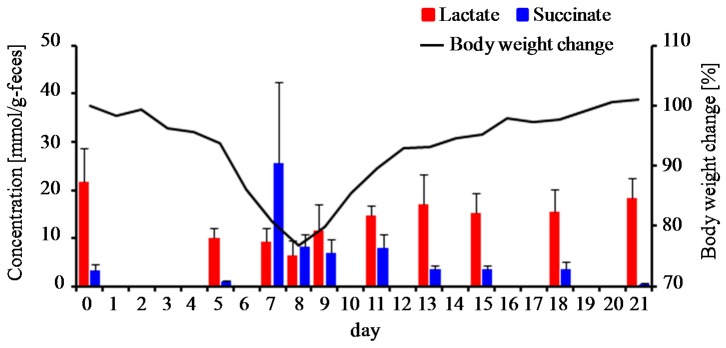
Monitoring of fecal lactate and succinate levels in DSS-treated mice. The solid lines show the average body weight change of five mice during the experimental period. Data of fecal lactate and succinate levels are mean ± standard error of the mean (SEM) (*n* = 5).

The changes in bacterial metabolite profiles can be inferred from the perturbation of gut microbiota caused by acute intestinal inflammation. In the present study, ^1^H-NMR analysis was employed to evaluate time-course profiles of fecal metabolites in DSS-treated mice. The PCA score plot of ^1^H-NMR data showed a fluctuation of gut metabolites of DSS-treated mice during the experimental period (Figure 4A). As a result, it was found that DSS treatment altered the signal intensity of several metabolites including essential amino acids (isoleucine, leucine, lysine, phenylalanine, tryptophan, and valine), non-essential amino acids (alanine, glutamate, glutamine, glycine, and tyrosine), succinate, and lactate (Figure 4B,C). In particular, the levels of succinate and lactate were elevated after the progressive phase. SCFAs (acetate, butyrate, and propionate) did not change in DSS-treated mice. In another animal experiment, time-course profiles of succinate and lactate in DSS-treated mice (*n* = 5) were re-evaluated with ion-chromatography and liquid chromatography with mass spectrometry, respectively (Figure 5). Fecal levels of succinate transiently increased around the peak of weight loss and decreased to the baseline levels in the recovery phase. On the other hand, fecal levels of lactate gradually decreased after the onset of colitis but increased to the baseline level in the recovery phase. Notably, PCA plots demonstrated that ^1^H-NMR-based metabolite profiles of mice recovered from DSS-induced colitis (at day 20) were similar with those of DSS-untreated mice (Figure 4A). Taken together, these results suggest the association of gut bacteria-derived metabolites, especially lactate and succinate, with the disease states of DSS-colitis mice.

### 3.4. Estimation of Gut Microbiota Associated with Succinate Production

We performed correlation analyses of gut microbiome (abundances of all OTUs) and ^1^H-NMR data (signal intensity of succinate) for screening the bacteria responsible for succinate production. A change in the fecal succinate level was positively correlated with seventeen OTUs (*r* > 0.5, *p* < 0.0004). Nine OTUs (*r* = 0.51–0.63) had 96.0–99.4% similarities with 16S rRNA sequence of *Lactobacillus intestinalis* (EU381126), four OTUs (*r* = 0.52–0.57) had 95.8–99.7% similarities with *Bacteroides caecimuris* (KR364741), two OTUs (*r* = 0.51–0.63) had 97.4% and 98.0% similarities with *B. thetaiotaomicron* (LC033799 and CP012937), one OTU (*r* = 0.51) had 97.6% similarity with *B. acidifaciens* (AB021159), and one OTU (*r* = 0.53) had 92.0% similarity with *Parasutterella excrementihominis* (LT558827).

## 4. Discussion

Our results on the microbiome during the colitis progressive phase demonstrate transient expansions of two specific bacterial families including the Bacteroidaceae and the Enterobacteriaceae. Previous studies have also reported the high abundance in the Bacteroidaceae in a DSS-induced colitis mouse model [8,42] and the colitogenic property of commensal *Bacteroides* species [43]. Deletion of commensal *Bacteroides* species by the metronidazole has been known to be effective for the prevention of the colitis development in DSS-treated mice [17,44]. These previous findings are consistent with our results that mice with high abundance of the Bacteroidaceae are highly sensitive to DSS. The expansion of Enterobacteriaceae is the common feature in experimental colitis models [8,45,46] and IBD patients [5,6], and may contribute to the perpetuation of intestinal inflammation [47]. Reactive oxygen species play an essential role in the pathogenesis of IBD [48]. Inflammation-mediated increases in intestinal oxygen levels may support the ecological niche of facultative anaerobic bacteria such as *Escherichia coli* [45] and the reduction of anaerobic bacteria including Bacteroidetes and Firmicutes in IBD animals [8,9] and patients [5,6]. Our results also demonstrate that some major anaerobic commensal bacteria (e.g., Lachnospiraceae, Ruminococcaceae, Bacteroidales S24-7 family) were greatly depleted after the onset of colitis. The members in the Lachnospiraceae and Ruminococcaceae play an essential role in the maintenance of gut immune homeostasis as inducers of colonic regulatory T cells [14,15]. It remains unclear whether the Bacteroidales S24-7 family contribute to the gut homeostasis because they have not yet been cultured. A recent metagenomic analysis described the ability of oxidative stress protection of the Bacteroidales S24-7 family, suggesting that they as well as *Bacteroides* species behave as anaerobes capable of growing under marginally oxic conditions [49,50]. Thus, it might be premature to ascribe the depletion of the Bacteroidales S24-7 family to changes in oxygen levels. Interestingly, Choo et al. (2017) also reported the depletion of the Lachnospiraceae and Ruminococcaceae and the increases in the Enterobacteriaceae and Lactobacillaceae in mice treated with vancomycin and imipenem [51], suggesting that they may be competitors with each other under non-inflammatory conditions. Further studies of how these commensal bacteria establish their ecological niches in the gut will help to illustrate the mechanisms for the improvement of the IBD-associated dysbiosis.

Time-course monitoring of fecal metabolites using ^1^H-NMR analysis revealed the association of organic acids (i.e., lactate and succinate) with the disease state of DSS-treated mice. Okada et al. (2013) reported that lactate produced by commensal lactobacilli is an essential trigger to induce the proliferation of intestinal epithelial cells (IECs) arrested by starvation [52]. Thus, we speculated that the elevated lactate levels associated with the remission affect the maintenance of IECs damaged by the exposure to DSS, although we have not yet obtained the direct evidence. Future studies focusing on the suppression of bacterial lactate production in the recovery phase using drugs (e.g., antibiotics, or lactate dehydrogenase inhibitors) are expected to provide new insights into the role of lactate in gut maintenance. Further, we observed a positive correlation (*r* > 0.5) between the succinate production and the relative abundances of some bacteria belonging to the genera *Bacteroides*, *Lactobacillus*, and *Parasutterella*. Some gut commensal bacteria, such as *Bacteroides* and *Prevotella*, have been reported as the producers of succinate [53]. However, the functional exploration of several genes associated with succinate biosynthesis, such as with a metagenomic approach, is required to precisely identify the succinate-producing bacteria in the recovery phase of DSS-induce colitis. 

Succinate has its pros and cons in relation to gut health. A recent report indicated *Bacteroides*-derived succinate plays an essential role in promoting the colonization of strict anaerobes such as Clostridia, which prevents the colonization and the growth of exogenous pathogens [54]. This might be due to the reduction in intestinal oxygen levels by the growth of aerobic and facultative anaerobic bacteria utilizing succinate. There may be the possibility that succinate supports the reconstitution of the gut bacterial ecosystem after colitis. As per its disadvantages, previous studies reported the contribution of *Bacteroides*-derived succinate to the development of colonic ulceration in DSS-treated mice [55], the exacerbation of enteric infection [56], and the expansion of *C. difficile* in the gut perturbed by antibiotics treatment or diarrhea [57]. Thus, further characterizations for judging whether the deletion of the succinate-producing bacteria is effective in protecting the colitis progression and secondary enteric infections are needed in future studies.

Another finding of ours is the characteristic changes in members of the genus *Lactobacillus* in DSS-treated mice. The onset of DSS-induced colitis leads a reduction in the abundance of *L. intestinalis* but no significant change in *L. johnsonii*, indicating different capacities for the adaptation to inflammation-mediated environmental changes among *Lactobacillus* species. On the other hand, the increased abundances of commensal *Lactobacillus* members seem to be associated with the recovery from colitis. Furthermore, the predominance of *Lactobacillus* members in the remission phase is accompanied by the regaining of the fecal metabolite profile, suggesting that the *Lactobacillus* promotes the improvement in the gut ecosystem. Their expansion may be attributed to the inflammation-mediated environment suitable for its growth. If this hypothesis is correct, the supplementation of *Lactobacillus*-containing probiotics in IBD animals and patients might confer beneficial effects such as dysbiosis correction and remission induction. Previous studies reported that a probiotic cocktail VSL#3 consisting of eight strains of lactic acid bacteria including four *Lactobacillus* species is effective in inducing the remission and manipulation of gut microbiota in experimental colitis animal [20] and IBD patients [18,19]. Establishing a further understanding of the ecophysiology of the commensal and probiotic *Lactobacillus* in the healthy or dysbiotic disease states will provide useful information for the successful treatment and prevention of chronic intestinal inflammation.

## 5. Conclusions

Chemical-induced colitis causes a transient expansion of colitogenic commensal bacteria, the depletion of beneficial commensal bacteria, and the fluctuation of intestinal lactate and succinate levels. Intestinal environments changed by host-mediated inflammation are favorable for the growth of commensal *Lactobacillus* members. These fundamental understandings of how intestinal inflammation impacts the gut ecosystem will provide the mechanistic explanation for and the correction of IBD-associated dysbiosis.

## Figures and Tables

**Table 1:** List of the top five most abundant operational taxonomic units (OTUs) in each phase. Taxonomic assignment of eleven OTUs was carried out with the basic local alignment search tool (BLAST) program in the DNA Data Bank of Japan (DDBJ) nucleotide sequence database.

OTU #	Closest Reference Strain (Acsession No.)	Identities	Ranking			
			Phase 1	Phase 2	Phase 3	Phase 4
414	*Lactobacillus johnsonii* strain UMNLJ22 (CP021704)	99.2%	1	1	3	1
527	*Bacteroides uniformis* strain CECT 7771 (AB021157)	97.7%		4		
629	*Escherichia coli* strain 4060 (FJ405333)	99.4%			2	
682	*Turicibacter* sp. LA62 (AB727349)	100%		5		
759	*Clostridium* sp. ARIAKE1333 (AB809059)	98.5%	5	3	4	3
826	*Lactobacillus murinus* (LC159538)	99.5%				2
1332	*Bacteroides caecimuris* strain I48 (KR364741)	99.4%				5
1387	*Escherichia coli* strain D1 (CP010134)	99.7%			5	
2045	*Bacteroides* sp. SLC1-38 (AB599946)	99.7%	4			
2095	*Bacteroides acidifaciens* (AB510696)	99.4%	2	2	1	
2301	*Lactobacillus intestinalis* strain ls74 (EU381126)	99.2%	3			4
